# Bempedoic Acid (ETC-1002): an Investigational Inhibitor of ATP Citrate Lyase

**DOI:** 10.1007/s11883-016-0611-4

**Published:** 2016-09-23

**Authors:** Ozlem Bilen, Christie M. Ballantyne

**Affiliations:** 1 Cardiovascular Research, Department of Medicine, Baylor College of Medicine, Houston, TX USA; 2 Cardiology, Department of Medicine, Baylor College of Medicine, Houston, TX USA; 3 Center for Cardiovascular Disease Prevention, Methodist DeBakey Heart and Vascular Center, Houston, TX USA

**Keywords:** ETC-1002, Low-density lipoprotein cholesterol

## Abstract

Bempedoic acid (ETC-1002), a novel therapeutic approach for low-density lipoprotein cholesterol (LDL-C) lowering, inhibits ATP citrate lyase (ACL), an enzyme involved in fatty acid and cholesterol synthesis. Although rodent studies suggested potential effects of ACL inhibition on both fatty acid and cholesterol synthesis, studies in humans show an effect only on cholesterol synthesis. In phase 2 studies, ETC-1002 reduced LDL-C as monotherapy, combined with ezetimibe, and added to statin therapy, with LDL-C lowering most pronounced when ETC-1002 was combined with ezetimibe in patients who cannot tolerate statins. Whether clinically relevant favorable effects on other cardiometabolic risk factors such as hyperglycemia and insulin resistance occur in humans is unknown and requires further investigation. Promising phase 2 results have led to the design of a large phase 3 program to gain more information on efficacy and safety of ETC-1002 in combination with statins and when added to ezetimibe in statin-intolerant patients.

## Introduction

Cardiovascular disease (CVD) is a major health problem and the leading cause of mortality and morbidity worldwide [1]. Alterations in lipid and lipoprotein metabolisms play an important role in pathogenesis of CVD. Low-density lipoprotein cholesterol (LDL-C)–lowering with statins inhibits progression of coronary atherosclerosis and reduces cardiovascular mortality and morbidity [2–4]. However, a large number of individuals still do not take high-efficacy doses of statins and continue to have elevated levels of LDL-C [5]. One of the major reasons for the lack of adherence to high- or moderate-dose statin therapy as recommended in the guidelines is the concern of both patients and physicians about adverse events with high-efficacy statins. The most common adverse events of statins are muscle-related adverse events, which range from myalgia to rare, but life-threatening, rhabdomyolysis [6], followed by asymptomatic elevation in hepatic transaminases [7]. Furthermore, recent evidence suggests that high-dose statins may increase the risk of developing type 2 diabetes [8]. Although such side effects are rare, they reduce patient compliance and medication adherence. Despite the proven benefits and wide availability of statins, because of their dose-dependent side effect profile, a considerable proportion of patients with elevated cholesterol fail to achieve guideline-recommended targets [9, 10]. This underlines the importance of developing alternative cholesterol-lowering therapies with good efficacy and tolerability beyond statins.

Bempedoic acid (ETC-1002) is one such novel cholesterol-lowering drug. It is an inhibitor of adenosine triphosphate (ATP) citrate lyase (ACL), a cellular enzyme responsible for production of precursors for fatty acid and cholesterol synthesis. ETC-1002 effectively reduces LDL-C and apolipoprotein (apo) B–containing lipoproteins [11••]. In this article, we will review the cardiometabolic effects of ETC-1002 and the underlying proposed mechanism of action in the light of the most recent evidence.

## ETC-1002 Mechanism of Action and Pharmacology

**Fig. 1:**
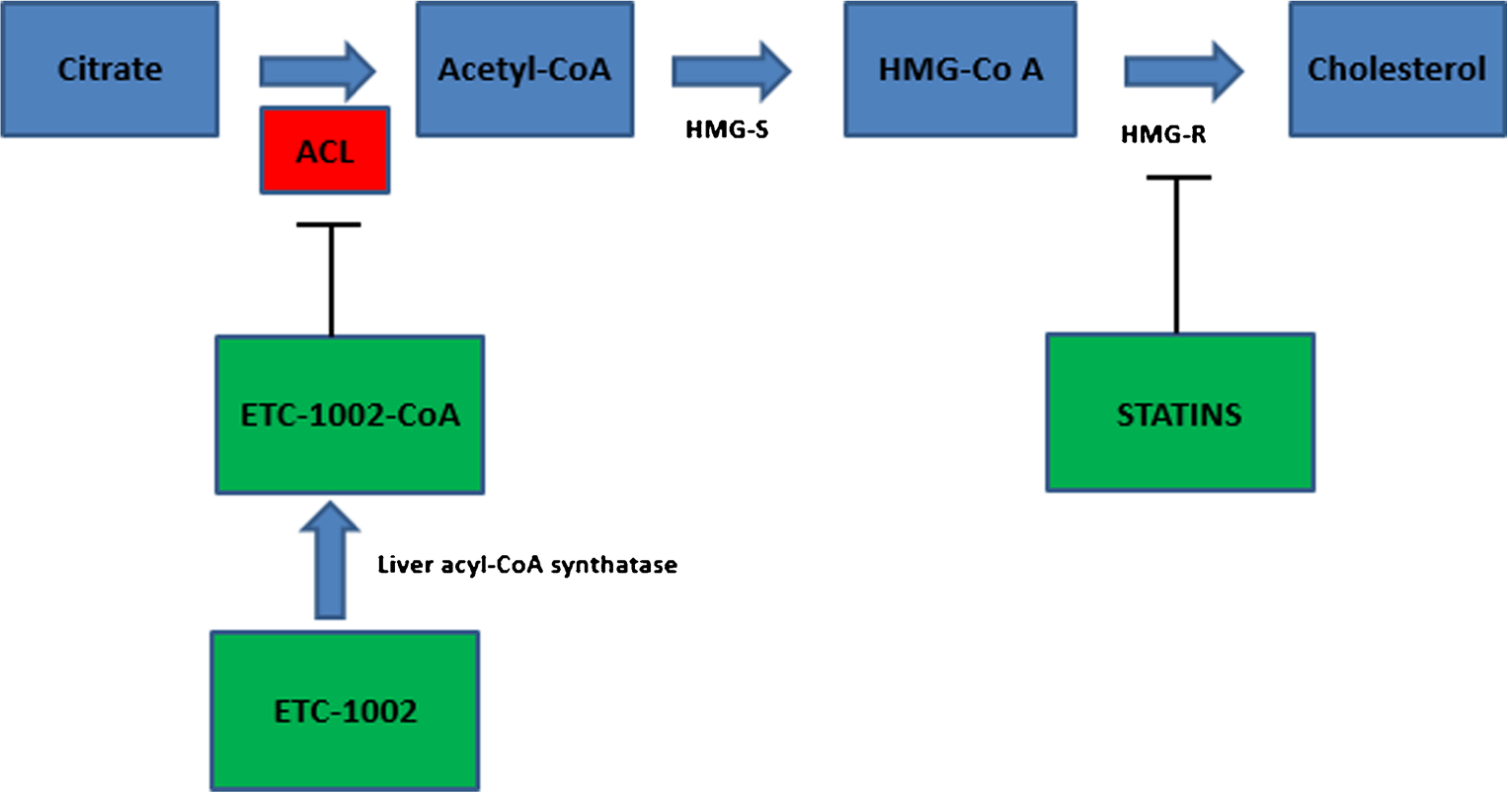
Major metabolic pathways affected by bempedoic acid (ETC-1002) in humans as supported by clinical trial data. Animal data suggested additional effects on triglyceride metabolism via ACL inhibition and other cardiometabolic pathways via AMP-activated protein kinase (AMPK) activation. *ACL* ATP citrate lyase, *Acetyl-CoA* acetyl coenzyme A, *HMG-S* HMG-CoA synthase, *HMG-R* HMG-CoA reductase

ACL is an important enzyme with significant effects on fatty acid and cholesterol metabolism. It is a cytosolic enzyme highly expressed in lipogenic tissues such as the liver and white adipose tissue [12] and is positioned upstream from HMG-CoA reductase in the mammalian cholesterol biosynthesis pathway [Fig. 1]. It links energy metabolism from carbohydrates to the production of fatty acids through catalyzing acetyl CoA synthesis, the fundamental substrate for the biosynthesis of both fatty acids and cholesterol [13, 14, 15••]. Its crucial role in lipid biosynthesis makes ACL a potential target for lipid-lowering intervention. Historically, several compounds have proven capable of inhibiting ACL in vitro, including (2),(2),2,2 difluorocitrate, several benzonesulfonamides, and the naturally occurring compound hydroxycitrate. Nevertheless, their development as pharmacologic agents has been limited due to poor ability to cross cell membranes, poor affinity for ACL, and poor specificity leading to undesirable inhibition of other essential enzymes in vivo. Among several ACL inhibitors that were tested, ETC-1002 (8-hydroxy-2,2,14,14-tetramethylpentadecaned–ioic acid; bempedoic acid) is in the most advanced stage of clinical development and has improved bioavailability and specificity compared with earlier compounds. It is a novel, oral, once-daily, small molecule with a half-life of 15–24 h. It is rapidly absorbed in the small intestine, and, importantly, the cell surface receptors through which this molecule enters the liver are different from statin transporters, thus there is no competitive liver uptake with statins [16]. ETC-1002 itself is a prodrug, which is converted to an active metabolite (ETC-1002–CoA) by endogenous liver acyl-CoA synthetase activity, which then inhibits ACL [Fig. 1]. By inhibiting cholesterol synthesis in the liver, ETC-1002 induces upregulation of the LDL receptor [17] and stimulates the uptake of LDL particles by the liver, which contributes to reductions of LDL-C levels in the blood [18]. In addition, because the prodrug is converted to the active drug specifically in the liver, theoretically this may avoid potential adverse muscle effects as seen with inhibition of cholesterol by statins in muscle. Although the precise mechanism of statin-induced myopathy is not agreed upon, cerivastatin had the highest systemic exposure (including muscle tissue) of all approved statins and the highest rate of myopathy. In animal studies, inhibition of ACL also led to reductions in fatty acids and triglycerides [19, 20].

Initial animal experiments showed that, in addition to inhibiting ACL, ETC-1002 also activates AMP-activated protein kinase (AMPK), a master kinase that regulates whole body energy metabolism and inhibits fatty acid and cholesterol synthesis pathways by inhibiting HMG-CoA reductase and acetyl-CoA carboxylase, the rate-limiting enzymes for cholesterol and fatty acid synthesis, respectively [20]. In a mouse model of diabetes and obesity, ETC-1002 improved hepatic steatosis; lowered plasma non-high-density lipoprotein cholesterol (non-HDL-C), triglycerides, and free fatty acids; and lowered glucose levels and improved glucose intolerance [21]. In addition, in human monocyte-derived macrophages treated with ETC-1002, it was shown that increased levels of AMPK phosphorylation reduced production of proinflammatory cytokines and chemokines [22]. Further, in a mouse model of diet-induced obesity, ETC-1002 restored adipose AMPK activity, reduced JNK phosphorylation, and diminished expression of macrophage-specific marker 4F/80. These data were consistent with decreased epididymal fat-pad mass and interleukin-6 release by inflamed adipose tissue [22]. Based on these studies, it was proposed that ETC-1002 may have potential benefits on systemic inflammation, glycemic control parameters, insulin resistance, and vascular complications of metabolic syndrome.

To date, ETC-1002 has been studied in more than 10 clinical trials across different patient populations [Table 1]. Although rodent studies suggested potential effects of ACL inhibition with ETC-1002 on both fatty acid and cholesterol synthesis, the clinical profile in humans shows an effect on cholesterol synthesis with no effect on fatty acid metabolism. Moreover, as discussed later in this article, although clinical studies showed a reduction in high-sensitivity C-reactive protein (hs-CRP) with ETC-1002, overall, there was a neutral effect on other cardiometabolic parameters such as weight, glucose metabolism, insulin resistance, and blood pressure, indicating that the effect of ETC-1002 on AMPK activation in humans is likely not clinically relevant.

**Table 1:** Bempedoic acid (ETC-1002) phase 1 and 2 clinical studies

Study number	Short title (*N* = total/ETC-1002 treated)	LDL-C lowering^a^	Dose range (mg)	Treatment duration (weeks)
001 [23]	Phase 1a in healthy subjects (*N* = 18)	–	–	Single dose
002 [24]	Phase 1b in healthy subjects (*N* = 53/39)	Up to 17 %	20, 60, 100, 120	2–4
003 [26••]	Phase 2a in patients with hypercholesterolemia (*N* = 177/133)	Up to 27 %	40, 80, 120	12
004 [25]	Phase 1b in healthy subjects (*N* = 24/18)	Up to 36 %	40, 180, 220	2
005 [27]	Phase 2a in patients with hypercholesterolemia and type 2 diabetes (*N* = 60/30)	43 %	80, 120	4
006 [31]	Phase 2a in patients with hypercholesterolemia and a history of statin intolerance (*N* = 56/37)	32 %	60, 120, 180, 240	8
007 [29]	Phase 2a in patients with hypercholesterolemia added on to atorvastatin 10 mg (*N* = 58/42)	22 %	60, 120, 180, 240	8
008 [32••]	Phase 2b in patients with hypercholesterolemia with or without statin intolerance versus ezetimibe (*N* = 349/249)	Up to 30 % (ETC-1002) Up to 48 % (ETC-1002+ ezetimibe)	120, 180, 120 + ezetimibe, 180 + ezetimibe	12
009 [30]	Phase 2b in patients with hypercholesterolemia while on stable statin therapy (*N* = 134/88)	Up to 24 %	120, 180	12
014 [31]	Phase 2a in patients with hypercholesterolemia and hypertension (*N* = 143/72)	21 %	180	6

^a^Average LDL-C % change from baseline

Another important approach to gain further insights into the effects of ACL inhibiton in humans would be to examine the association between genetic variants and metabolic parameters and cardiovascular outcomes in large epidemiological studies as has been done recently for a number of potential targets for lipoprotein metabolism.

## Phase 1 Studies

A first-in-human, phase 1a single-dose clinical trial, *ETC-1002-001* [23] evaluated safety, tolerability, and pharmacokinetics of ETC-1002 in 18 healthy subjects. Similarly, *ETC-1002-002* [24] was a staged 2-week and 4-week phase 1b multiple dose tolerance clinical trial in 53 subjects, with 39 receiving ETC-1002 and 23 receiving placebo. The subjects were divided into four different cohorts of six subjects with each receiving 20, 60, 100, or 120 mg of ETC-1002 or placebo once daily for 14 days. This was followed by studying a larger cohort that was treated for 28 days during which subjects lived outside of the clinical site for the duration of their treatment. ETC-1002 was safe, well tolerated, and associated with no dose-limiting side effects.

Finally, *ETC-1002-004* [25] was a 2-week, phase 1b, multiple dose tolerance clinical trial in 24 subjects, of whom 18 received ETC-1002. This clinical trial was designed to evaluate the safety and tolerability of escalating, multiple oral doses of ETC-1002 above 120 mg/day. Subjects in this clinical trial received 140, 180, or 220 mg of ETC-1002 or placebo once daily for 14 days. LDL-C levels were reduced by an average of 36 % for subjects receiving 220 mg/day of ETC-1002 as compared to a 4 % increase for subjects receiving placebo (*p* < 0.0001). No serious adverse events were observed in the subjects dosed with ETC-1002. ETC-1002 was safe, well tolerated, and associated with no dose-limiting side effects.

## Phase 2 Studies

### ETC-1002 as Monotherapy in Patients with Hypercholesterolemia

The first phase 2 study, *ETC-1002-003* [26••], was a 12-week phase 2a proof-of-concept study in 177 patients, of whom 133 were treated with ETC-1002, across 11 participating clinical recruitment sites in the USA. This clinical study was designed to evaluate the LDL-C–lowering efficacy and safety of ETC-1002 versus placebo in patients with hypercholesterolemia (LDL-C of 130 to 220 mg/dL) and either normal triglycerides (less than 150 mg/dL) or elevated triglycerides (150 to 400 mg/dL). The four arms were placebo and 40-, 80-, and 120-mg doses of ETC-1002 once daily. LDL-C levels were reduced by an average of 18, 25, and 27 % for patients treated with ETC-1002 40, 80, and 120 mg, respectively, compared with an average of 2 % for patients treated with placebo (*p* < 0.0001). ETC-1002’s lowering of LDL-C levels was maintained across a range of baseline triglyceride levels. ETC-1002 also lowered levels of the atherogenic biomarkers apo B, non-HDL-C, and LDL particle number (*p* < 0.0001) in a dose-dependent manner. Patients treated with ETC-1002 demonstrated a trend in hs-CRP reduction of 20 to 26 % compared with 2 % in patients treated with placebo. In a subgroup of patients with elevated hs-CRP, patients treated with ETC-1002 demonstrated a trend in hs-CRP reduction of 43 to 64 %, compared with a decrease of 7 % in patients treated with placebo.

*ETC-1002-005* [27] was a 4-week phase 2a proof-of-concept clinical study at a single site that was designed to evaluate the LDL-C–lowering efficacy and safety of ETC-1002 in patients with type 2 diabetes. One treatment arm was placebo and the other was 80 mg of ETC-1002 once daily for 2 weeks followed by 120 mg of ETC-1002 once-daily for 2 additional weeks. LDL-C levels after 4 weeks of treatment with ETC-1002, which was the primary endpoint, were reduced by an average of 43 % in patients receiving the 120-mg dose of ETC-1002, compared with an average of 4 % in patients receiving placebo (*p* < 0.0001). Approximately 80 % of the patients were not at their National Cholesterol Education Program Adult Treatment Panel III LDL-C goal of less than 100 mg/dL at the beginning of the study. Of these, 88 % of the patients treated with ETC-1002 achieved their goal by study end as compared with 4 % of patients treated with placebo (*p* < 0.0001). Levels of hs-CRP were reduced by 41 % with the 120-mg dose of ETC-1002 versus 11 % with placebo (*p* = 0.001). Non-HDL-C decreased by 32 % in patients treated with ETC-1002 as compared with an increase of 1 % in patients treated with placebo (*p* < 0.0001). A 24-h continuous glucose monitoring assessment showed a non-significant trend of improved glycemic control with ETC-1002 treatment. Overall, 24-h ambulatory blood pressure monitoring showed no differences between treatment groups in mean changes from baseline to day 28.

Finally, *ETC-1002-014* [28] was a 6-week, multicenter, randomized, double-blind, placebo-controlled, parallel group phase 2 study that evaluated the safety and efficacy of ETC-1002 versus placebo in 143 patients with both hypercholesterolemia and hypertension. After washout of any lipid-modifying and blood pressure therapies, 71 patients received ETC-1002 180 mg and 72 patients received placebo. ETC-1002-treated patients achieved LDL-C lowering of 21 % at 6 weeks, compared with an increase in LDL-C of 3 % in the placebo group (*p* < 0.0001). The reduction occurred within the first 2 weeks of initiating therapy and continued throughout the treatment period. Levels of hs-CRP were reduced by 25 % with ETC-1002, compared with an increase of 20 % in the placebo group (*p* < 0.0001). ETC-1002 had a neutral effect on blood pressure and was safe and well tolerated. Despite effective LDL-C lowering with ETC-1002, HDL-C and triglyceride levels were unchanged across all treatment arms in these studies. No serious adverse events were observed in patients treated with ETC-1002. ETC-1002 was safe, well tolerated, and associated with no dose-limiting side effects.

### ETC-1002 Added on a Background of Statin Therapy in Patients with Hypercholesterolemia

*ETC-1002-007* [29] was an 8-week phase 2a clinical study in 58 patients, of whom 42 were treated with ETC-1002, across six clinical recruitment sites in the USA. For the primary endpoint of number of subjects with adverse events, clinical lab abnormalities, and other safety findings, ETC-1002 as an add-on to 10 mg of atorvastatin was well tolerated and did not result in any serious adverse events. Although the trial was not designed to assess LDL-C lowering with ETC-1002, this was measured as a secondary endpoint to determine whether incremental LDL-C lowering would occur with ETC-1002 added on a background of statin therapy. In patients on a background of atorvastatin, ETC-1002 reduced LDL-C levels by an average of 22 versus 0 % change with placebo (*p* < 0.0001).

*ETC-1002-009* [30] was a double-blind, parallel-group, placebo-controlled multicenter trial that evaluated 134 patients with baseline LDL-C of 115–220 mg/dL while taking atorvastatin ≤20 mg, simvastatin ≤20 mg, rosuvastatin ≤10 mg, or pravastatin ≤40 mg randomized to receive ETC-1002 120 mg, ETC-1002 180 mg, or placebo once daily for 12 weeks. ETC-1002 lowered LDL-C by up to 24 % (*p* < 0.0001), significantly more than placebo, as an add-on to statin therapy. ETC-1002 also lowered (*p* < 0.05) apo B, non-HDL-C, and total cholesterol levels and LDL particle number by more than placebo. Non-significant reductions in hs-CRP were observed with ETC-1002 120 mg (22 %, *p* = 0.26) and 180 mg (30 %, *p* = 0.08) versus 0 % with placebo. No significant changes in HDL-C or triglyceride levels were observed in either study. Adverse events, muscle-related adverse events, discontinuations due to adverse events, and levels of clinical safety labs were generally similar compared with placebo.

Overall, the LDL-C–lowering effect of ETC-1002 added on background statin therapy appeared to be less pronounced as compared to ETC-1002 monotherapy. This is likely due to the overlapping mechanism of action of both drugs [Fig. 1]. Whether the additive cholesterol-lowering effect of ETC-1002 would be less with higher dose background statin therapy will require further investigation in phase 3 studies.

### ETC-1002 as Monotherapy and in Combination with Ezetimibe in Patients with Statin Intolerance

*ETC-1002-006* [31] was an 8-week phase 2a proof-of-concept clinical study in 56 patients, of whom 37 were treated with ETC-1002, across five clinical recruitment sites in the USA. This clinical study was designed to evaluate the LDL-C–lowering efficacy, tolerability, and safety of ETC-1002 versus placebo in patients with hypercholesterolemia and a history of intolerance to one or more statins, defined as new myalgia, muscle cramps, muscle aches, or muscle weakness that developed during statin treatment and resolved or markedly improved of muscle symptoms within 4 weeks of statin discontinuation. After completing a washout of lipid-lowering therapy and 2 weeks of treatment with placebo, eligible patients were randomized to receive ETC-1002 or placebo in a 2:1 ratio for 8 weeks. Patients were given either increasing doses of ETC-1002 of 60, 120, 180, and 240 mg for 2 weeks each or placebo only for the full 8 weeks. The primary endpoint of this clinical study was LDL-C lowering from baseline to end of study. LDL-C levels after 8 weeks of treatment with ETC-1002 were reduced by an average of 32 % in patients treated with ETC-1002, compared with an average of 3 % in patients treated with placebo (*p* < 0.0001). Drop-out rates and muscle-related adverse events were comparable between ETC-1002 and placebo, and no patients treated with ETC-1002 discontinued the trial because of muscle-related adverse events. hs-CRP levels were reduced by 42 % after 8 weeks of ETC-1002 therapy versus 0 % on placebo (*p* = 0.0022). No significant changes in HDL-C or triglyceride levels were observed.

*ETC-1002-008* [32••] was a phase 2b study of the treatment of elevated LDL-C levels in approximately 348 patients either with (*n* = 177) or without (*n* = 171) statin intolerance across 70 clinical sites in the USA. The purpose was to assess the dose response of ETC-1002, directly compare the LDL-C–lowering efficacy of ETC-1002 versus ezetimibe, and assess safety and tolerability, including muscle-related adverse events, in patients with or without statin intolerance, defined as inability to tolerate two or more statins because of muscle pain, weakness, or cramping that began or increased during statin therapy and resolved with statin discontinuation, including at least one statin at the lowest approved daily dose (rosuvastatin 5 mg, atorvastatin 10 mg, simvastatin 10 mg, lovastatin 20 mg, pravastatin 40 mg, fluvastatin 40 mg, pitavastatin 2 mg) or less. With a parallel-group design and 12-week duration, ETC-1002-008 compared two doses of ETC-1002 (120 and 180 mg) with ezetimibe, a common treatment in patients with statin intolerance. The LDL-C–lowering efficacy of ETC-1002 in combination with ezetimibe was also assessed. In patients receiving ETC-1002 monotherapy, compared with patients receiving ezetimibe monotherapy, significantly greater LDL-C lowering of up to 30 % was observed. LDL-C reduction occurred within the first 2 weeks of treatment and was sustained over the treatment period. ETC-1002 lowered LDL-C similarly in both statin-intolerant and statin-tolerant patients. Lowering of atherogenic lipids and lipoproteins was consistent with LDL-C lowering. Significantly more hs-CRP lowering of up to 40 % was seen with ETC-1002 compared with ezetimibe. In patients receiving both ETC-1002 and ezetimibe, LDL-C lowering of up to 48 % was observed and the combination appeared to be safe and well tolerated. HDL-C decreased with ETC-1002 treatment by up to 6 % and increased with ezetimibe alone by 5 % (*p* < 0.0001 to *p* < 0.05 for ETC-1002 groups vs. ezetimibe alone). In patients treated with ETC-1002, including those with statin intolerance, there were no increases in muscle-related adverse events as compared with ezetimibe.

Overall, ETC-1002 and ezetimibe in combination seems to be a safe and effective regimen in patients with statin intolerance. Long-term studies are needed to assess the effect of this combination on cardiovascular outcomes.

## Conclusion

Currently, available results of clinical trials suggest that bempedoic acid (ETC-1002) may represent a novel therapeutic approach for LDL-C lowering. In seven phase 2 studies which randomized a total of 977 participants of whom 669 received active drug, ETC-1002 has been shown to reduce LDL-C as monotherapy, combined with ezetimibe, and added to statin therapy, with LDL-C lowering most pronounced when ETC-1002 was combined with ezetimibe in patients with a history of statin intolerance. Although rodent studies had suggested potential effects of inhibition of ACL on both fatty acid synthesis and cholesterol synthesis, the clinical profile in humans is consistent with a major effect on cholesterol synthesis but not on fatty acid synthesis. Whether clinically relevant favorable effects on other cardiometabolic risk factors such as hyperglycemia and insulin resistance occur in humans is unknown and requires further investigation in studies that are primarily designed to test such effects. The current results on efficacy and tolerability have led to the design of a large phase 3 program to gain more information on both efficacy and safety in combination with statins and also when added to ezetimibe in statin-intolerant patients.
